# Construction of a large-scale semi-field facility to study genotypic differences in deep root growth and resources acquisition

**DOI:** 10.1186/s13007-019-0409-9

**Published:** 2019-03-20

**Authors:** Simon Fiil Svane, Christian Sig Jensen, Kristian Thorup-Kristensen

**Affiliations:** 1 Department of Plant and Environmental Science, University of Copenhagen, 1871 Frederiksberg, Denmark; 2 Research Division, DLF Seeds A/S, 4660 Store Heddinge, Denmark

**Keywords:** Drought, Minirhizotron, Nitrogen, Phenotyping, Root, Semi-field, Soil, Water

## Abstract

**Background:**

Roots are vital organs for plants, and the effective use of resources from the soil is important for yield stability. However, phenotypic variation in root traits among crop genotypes is mostly unknown and field screening of root development is costly and labour demanding. As a consequence, new methods are needed to investigate root traits of fully grown crops under field conditions, particularly roots in the deeper soil horizons.

**Results:**

We developed a new phenotyping facility (RadiMax) for the study of root growth and soil resource acquisition under semi-field conditions. The facility consists of 4 units each covering 400 m^2^ and containing 150 minirhizotrons, allowing root observation in the 0.4 m–1.8 m or 0.7 m–2.8 m soil depth interval. Roots are observed through minirhizotrons using a multispectral imaging system. Plants are grown in rows perpendicular to a water stress gradient created by a multi-depth sub-irrigation system and movable rainout shelters. The water stress gradient allows for a direct link between root observations and the development of stress response in the canopy.

**Conclusion:**

To test the concept and technical features, selected spring barley (*Hordeum vulgare* L.) cultivars were grown in the system for two seasons. The system enabled genotypic differences for deep root growth to be observed, and clear aboveground physiological response was also visible along the water stress gradient. Although further technical development and field validation are ongoing, the semi-field facility concept offers novel possibilities for characterising genotypic differences in the effective use of soil resources in deeper soil layers.

**Electronic supplementary material:**

The online version of this article (10.1186/s13007-019-0409-9) contains supplementary material, which is available to authorized users.

## Background

Effective use of water and nutrients is important to ensure a sustainable crop production. In Europe future droughts are expected to set in more quickly and be more intense [1, 2]. At the same time, new measures are needed to reduce the nitrogen (N) pollution of the environment [3]. During dry conditions, the ability of deep and vigorous root growth to utilize deep subsoil water is important [4–6]. Furthermore, acquisition of leached N in deeper soil layers could improve nitrogen use efficiency (NUE) and reduce N losses to the environment [7–9].

Plant breeders have largely focused on shoot parameters as they seek new plant varieties that have better yield potential and other desirable qualities. Considerable effort is needed to obtain information about the entire root system, since most crop species extend their roots to soil layers deeper than 1 m [10]. Excavations of complete root system have been attempted [11, 12] and has provided data regarding overall root system architecture. Auger sampling followed by root washing and trench wall methods has provided some relief [13]. Over time, further attempts to reduce workload range from field-based methods on mature root systems to more indirect methods of early root growth in greenhouse systems (controlled environment).

For field phenotyping, the core break method [14, 15] and minirhizotron (MR) method [7, 16, 17] have become widely used alternatives to root studies made by complete excavations. Neither methods require time-consuming procedures for washing roots that have been extracted from soil samples. MR studies offer the further advantage of repeated measurements over time. On the other hand, MR studies are often hampered by soil smearing during tube installation, poor tube-to-soil contact and overestimation of deep root intensities if roots grow preferentially along the tube surface [18–21]. However, by careful horizontal insertion techniques, such artifacts can be reduced [19]. However, both core break and MR techniques suffer from a time-consuming bottleneck: the subsequent manual quantification of root structures.

Other methods in use focus on indirect but easily quantifiable “proxy traits”. Methods such as the “Shovelomics” approach, have been used to identify genetic differences in larger populations of maize [22]. Some methods combine newly developed and low-cost Unmanned Aerial Systems (UAS-imaging) to identify drought-related stress symptoms [23]. Finally, multiple proxy-trait screening systems offer rapid detection under controlled greenhouse conditions e.g. [24–28]. Field tests have shown a good correlation between proxy traits such as seminal root angle and performance in the field, but the direction and magnitude of correlations varied across environments [29, 30]. To improve detection and validation of desirable proxy traits, a screening system can be run in parallel to field validation in different target environments as suggested by [5]. Thus, new methods to study the actual deep root development of crops grown to their full development in field conditions, will be beneficial both for validation of existing methods and improve the understanding of the relationships between proxy traits and actual root development in deeper soil horizons.

Here we describe a new phenotyping infrastructure for the identification of plant material capable of utilizing water and nutrients in deeper soil layers. The semi-field approach allows for a high degree of control of soil and climatic factors between seasons, but in an environment that closely resembles field conditions. Furthermore the facility is large enough to include many genotypes having 300 rows available for each experiment. Our facility enables root measurements to be coupled directly with observations of canopy drought stress symptoms induced by controlled water stress. This paper includes results from a 2-year replicated spring barley experiment to illustrate the design and function.

## Materials and methods

### Outline of the facility

**Fig. 1:**
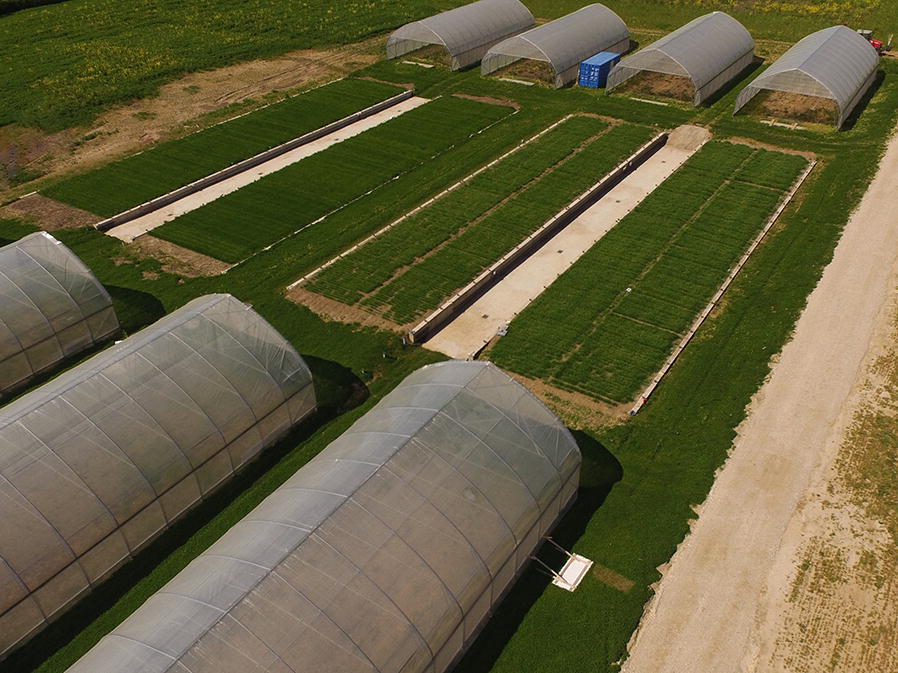
Overview of the phenotyping facility in May 2017. Spring barley was established in units 1 and 2 (front). Perennial ryegrass was grown in units 3 and 4 (photo by Jesper Svensgaard, 2017)

**Fig. 2:**
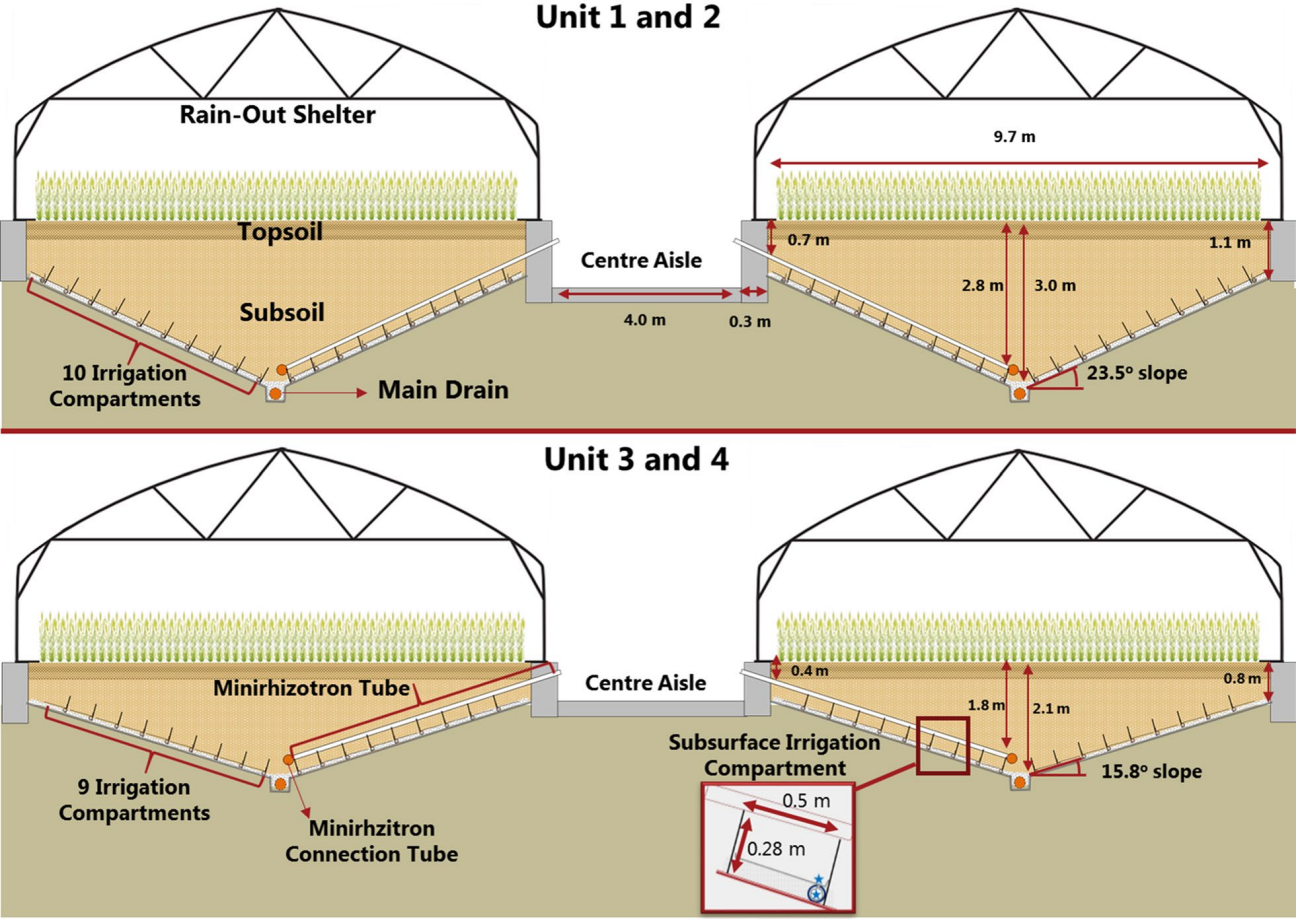
Cross-section of the four units in the RadiMax facility

The RadiMax facility is located at Copenhagen University’s experimental farm (Latitude 55.66815°N, Longitude 12.30848°E), west of Copenhagen, Denmark and at an elevation of 26 m above sea level (Fig. 1). The experimental space includes four units each with a net area of 9.7 × 40 m. The units comprise two pairs consisting of two units each connected by a 4-m center aisle (Fig. 2). Each unit has concrete walls and a V-shaped bottom lined with an impermeable plastic membrane. The soil profile consists of repacked soil with two distinct soil layers, topsoil 0–0.4 m with subsoil below. One pair of units (1 and 2), designed for deep-rooted crops, has a soil depth that increases from 1.1 m at the sides to 3.0 m in the middle (Fig. 2), while soil depth in the other pair ranges 0.8 m to 2.1 m.

**Fig. 3:**
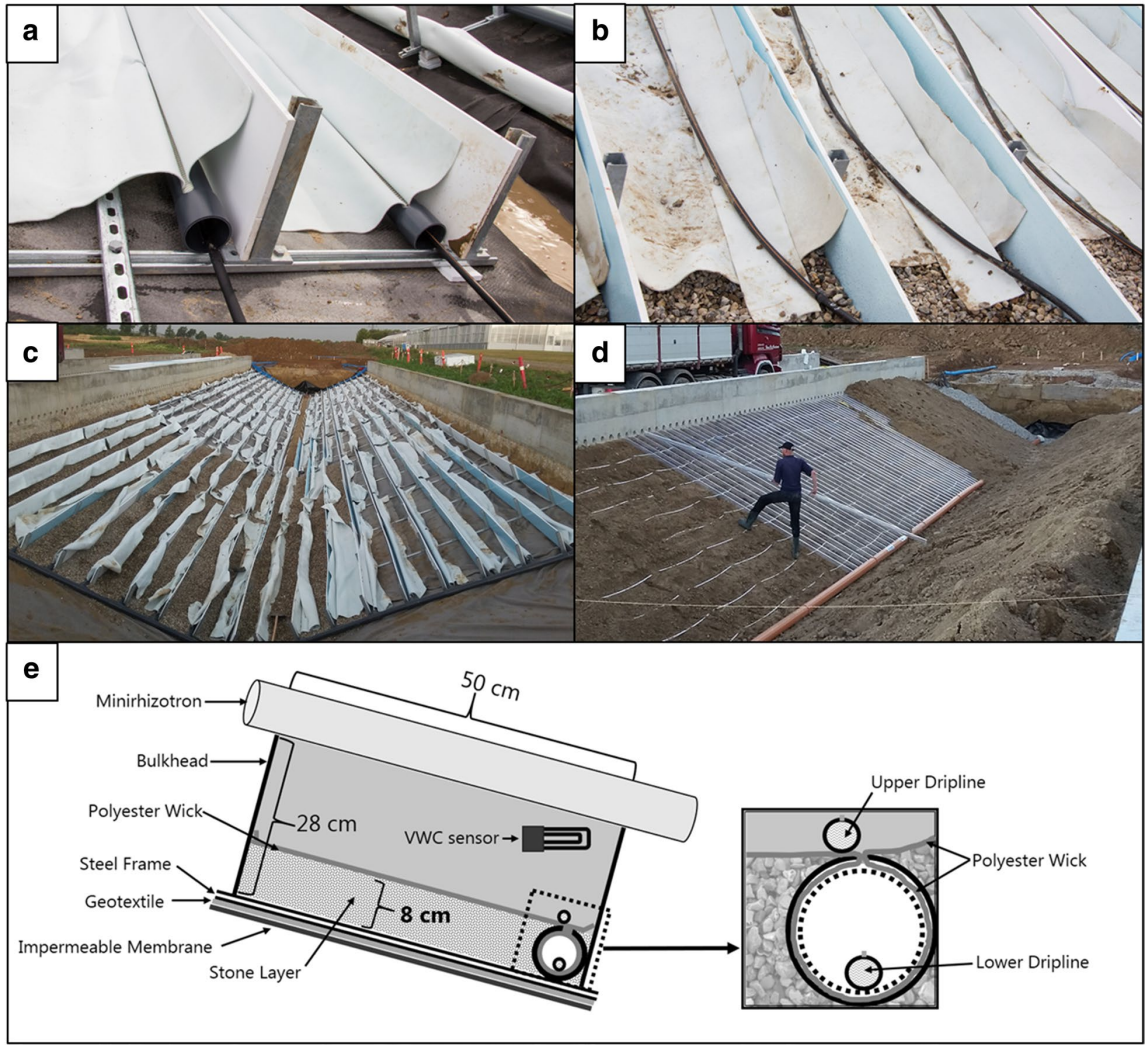
Installation of the subsurface irrigation compartments and MR tubes. **a** Irrigation tube lined with wicking material supported by a steel frame skeleton. Lower driplines placed within the tube. **b** Irrigation tube fixed by stone fill with upper dripline installed before soil backfilling. **c** Overview of subsurface irrigation compartments during installation. **d** Installation of MR tubes after soil backfilling. **e** Schematic drawing of the subsurface irrigation compartments

Transparent tubes for root observations (minirhizotrons, MR) were installed 40 cm above the sloping bottom. At one end, the tubes were led through holes in the concrete wall that faces the central aisle; at the other end, the tubes were led towards a central connection tube above a central drain (Figs. 2, 3d). The MR tubes thus enable photography of roots ranging from a soil depth of 0.70 m to 2.70 m for units 1 and 2, and from 0.40 m to 1.80 m for units 3 and 4. A total of 600 MR tubes (PMMA-Plastic) with a 70 mm outer diameter, 60 mm inner diameter and a total length of 5.5 m were placed, 25 cm apart, in the four units (150 in each).

Two moveable rainout shelters were constructed for each unit, making it possible to cover the units in rainy periods (see Fig. 1). A steel frame of 22 m × 10 m × 4.9 m (L × W × H) forms a tunnel greenhouse with straight sides (1.75 m), type RS 99300 (Rovero, Ramsdonksveer, NL). The roof of the shelters is constructed with three layers of plastic film (TPT, Solar Eva 5) with a light transmission of 91%. The open ends provide ventilation and a transparent insect net (Mesh size 0.39 mm × 0.88 mm) was used as wall cladding to allow airflow and reduce warming effects, but still prevent rain from entering from the side. When not in use, the shelters are parked 10 m to the east and west of the units (Fig. 2).

### Subsurface irrigation system

A water supply gradient was created by a subsurface irrigation system placed at the bottom of each unit. After water has been emitted from driplines placed at the bottom, the system uses the principle of capillary movement to distribute water without the assistance of external forces (Fig. 3e). Premade irrigation tubes (Ø 70 mm) were installed in the bottom of each compartment. Each tube is open along its upper side having a coating of polyester textile (Breatex450, Fibertex, Aalborg, Denmark) providing direct capillary contact with the soil above. By placing the polyester textile above a 10 cm layer of prewashed stones (16–32 mm) a capillary barrier is formed between the soil and the bottom (Fig. 3). The compartments are separated by 0.28 m tall bulkheads of steel-supported PVC plates, installed at a 90 ° angle to the bottom and at a distance of 50 cm (Fig. 3c). Bulkheads reduce horizontal flow and function as support for the MR tubes. To allow for release of water over the entire length of the facility, a pressure-compensated dripline system was used (UniRam™ HNCL (Netafim, Tel Aviv, Israel). Each compartment contains two driplines. One dripline was placed within the irrigation tube and the other directly above the tube in the interlayer between the soil and the wick material. Each dripline supplies water from drippers placed 0.20 m apart, with a total dripper flow rate of 0.85 L h^−1^. During irrigation, the water table (pF = 0; pF = log|cm H_2_O|) is defined at the bottom of each tube controlled by the capillary flow by the polyester wick. A slope of 6.7‰ along the length of the facility ensure drainage of excess water.

**Fig. 4:**
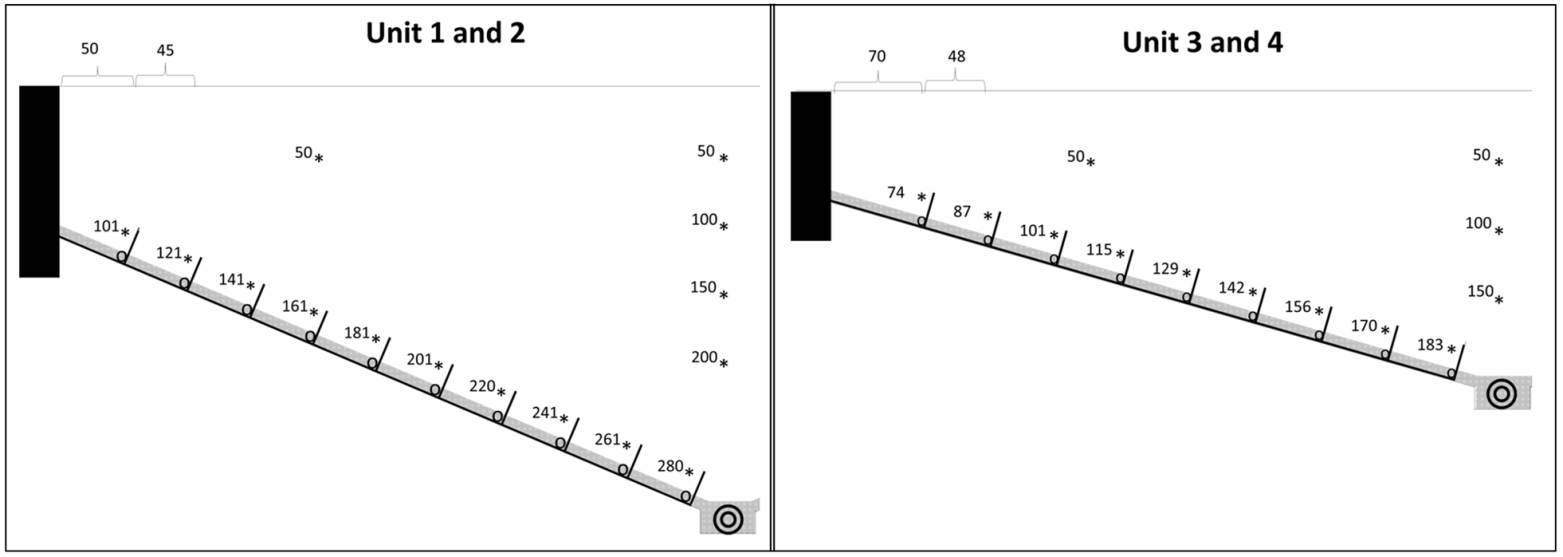
Schematic illustration of the position of the volumetric water content sensors

A sensor system was installed within the soil profile and irrigation compartments. The sensor network consists of TDT volumetric water content (VWC) and temperature sensors (Acclima, Inc., Boise, ID, USA). Volumetric water content is determined following the factory calibration using the Topp equation [31]. VWC sensors were placed at two positions in the middle of each unit (West and East) at 0.5 m depth increments (Fig. 4). Furthermore, VWC sensors installed 0.25 m above the bottom provides a measurement within each irrigation compartment. Two CR6 data loggers (Campbell Scientific, INC., UT, USA) collect sensor data at 5 min intervals.

### Soil properties

Two sandy loam soil mixes, a nutrient-rich topsoil mix and nutrient-poor subsoil mix, were prepared using locale soil resources (Table 1). The topsoil was excavated on-site from the A-horizon prior to construction. A subsoil mix was made from the B- and C-horizons (0.5 m–2 m) from a field located 12 km from the experimental farm, but of similar origin as the topsoil. The subsoil was excavated under dry conditions in August 2015, following a rapeseed crop. The top- and subsoil were mixed separately, and stones larger than 25 mm were removed by screening (Powerscreen Warrior, 1400). The soil mixes were carefully repacked into the four units during periods of dry weather, one unit at a time, between August and October 2015. The soil was exposed to autumn and winter precipitation while it settled. 12 months later, a final 10 cm of topsoil was added, making the total topsoil depth 0.4 m.

**Table 1:** Soil texture of the fine soil fraction (< 2 mm), mass fraction of gravel (2–25 mm), soil bulk density, field capacity (FC), permanent wilting point (PWP), porosity and chemical composition of the top and subsoil of the facility

	Soil depth	Clay	Silt	Fine sand	Coarse sand	Organic matter
		< 2ɥm	2–50 ɥm	50–500 ɥm	500–2000 ɥm	
	m			%		
Topsoil	0–0.4	12.0 (0.2)	13.0 (0.7)	44.7 (0.7)	28.5 (1.0)	1.7 (0.10)
Subsoil	0.5–1.0	13.1 (0.4)	13.2 (0.7)	45.7 (1.1)	27.5 (0.4)	0.5 (0.05)

	Soil depth	Gravel	Bulk density	FC	PWP	Porosity
	m	%	g cm^−3^		vol%	
Topsoil	0–0.4	2.0	1.59 (0.01)	24	6	40
Subsoil	0.5–1.0	8.7	1.72 (0.02)	20	12	35

	Soil depth	P	K	Mg	pH_(0.01 M CaCl2)_	
	m		mg kg^−1^			
Topsoil	0–0.4	3.8 (0.2)	11.1 (0.9)	5.1 (0.1)	7.0 (0.04)	
Subsoil	0.5–1.0	0.8 (0.1)	3.5 (0.1)	2.9 (0.1)	7.5 (0.05)	

Mean value of the four units with standard error in brackets, n = 4

Soil samples were collected using a 1.5 m soil core sampler. Ten subsamples were collected from a cross-section at each end of the units and divided into two depth intervals 0–0.3 m and 0.5–1.0 m. Soil particle size distribution of the fine soil (< 2 mm) was measured by sieving and sedimentation following [32]. Soil Organic carbon, pH and the available content of P, Mg and K were measured using plant available extraction methods that are standard in Denmark [33]. The mass fraction of gravel was measured in only one of the four units. In March 2017 the soil bulk density was measured by sampling of soil cores at 0.25 m and 0.7 m with two samples collected from each unit. Each sample consisted of 10 subsamples (50 mm height, 50 mm diameter) and oven-dried to a constant weight at 105 °C. The soil physical properties were measured In-situ by sensors installed within the repacked soil matrix (see Additional file 1). Detailed measurements of soil texture of the fine soil, bulk density and chemical composition within each unit are available (Additional file 2).

## Experimental design and measurements

Selections of European spring barley (*Hordeum vulgare* L.) lines were grown in two seasons in a complete randomized block design. The majority of these are modern breeding lines. However, to allow for replicated measurement over the two seasons, seven marketed cultivars were selected on the basis of shoot morphological characteristics and year of release. Three of these (Laurikka, Invictus and Evergreen) are modern cultivars characterized by high yield in conventional crop production in northern Europe, while a fourth, Evelina, is aimed for organic growth conditions in central Europe. The remaining cultivars (Tocada, Prisma, and Kenia) were selected because previous work shows contracting differences in NUE [34].

The spring barley lines were seeded in rows perpendicular to the center aisle and directly on top of the MR tubes below, giving a 25 cm row distance. Dates and details for crop establishment, management and sampling are presented in Table 2. In 2016, 59 lines were grown in two replicated rows in unit 3, while the 2017 experiment had 74 lines grown in four replicated rows within units 1 and 2. Data of Grain yield and protein content was gathered for all cultivars in both seasons. At harvest, each row was divided into four subsamples: two from the deep mid-section (area_2016_ = 2.23 m × 0.25 m, area_2017_ = 2.13 m × 0.25 m) and two from the border section (area_2016_ = 2.13 m × 0.25 m, area_2017_ = 1.93 m × 0.25 m) of each unit. The outermost 0.5 m was defined as border and removed before harvest. At harvest, ears were collected, dried to constant weight, threshed and weighed. Grain protein content and water content was determined by near-infrared transmission measurement (Intratec grain analyzer, Foss, Hilleroed, Denmark). Grain nitrogen content was estimated on the basis of the grain protein content using 6.25 as the conversion factor for barley [35].

**Table 2:** Overview of the two experiments

Treatment	Exp. 1	Exp. 2
Unit	3	1 and 2
Previous crop	Rye (cover crop)	Wheat/grass (+ cover crop)
Plowing	23.03.16	20.12.16
Cultivars	59 (n = 2)	76 (n = 4)
Cultivars test experiment (rows)	7 (n = 4)	7 (n = 4)
Seeding date	12.04.16	28.03.17
Seeding density (seeds m^−2^)	300	300
Fertilizer date	12.04.16	28.03.17
Fertilizer application rate (kg N ha^−1^)	70	100
Root imaging	03.06.16/28.06.16	05.06.17/23.06.17
Day of flowering (anthesis)	≈ 13.06.16	≈ 14.06.17
Rainout shelter deployed	15.06.16	07.06.17
Harvest	02.08.16	03.08.17

The seven marketed cultivars were grown in both seasons in four replicated rows. For these rows, data of grain yield and protein were supplemented with measurement of total nitrogen content and ^13^C enrichment (2017 only), but only for half of the row (the part of the row that contained MR tubes (i.e., from the concrete aisle to the centerline, see Fig. 2). Samples consisting of one ear sampled from each of eight different plants were taken from four positions (1 m intervals starting 0.5 m from the border). The samples were dried at 75 °C for 48 h, weighed, milled, before total carbon, nitrogen and ^13^C were measured using a PDZ Europa ANCA-GSL elemental analyzer interfaced to a PDZ Europa 20-20 isotope ratio mass spectrometer (Sercon Ltd., Cheshire, UK).

Agrometeorological data were acquired from a weather station located 600 meters west of the facility. Reference Evapotranspiration (ET_o_) was calculated hourly by the FAO-Penman–Montieth method using measured data of Air Temperature (Z = 2), Air humidity (Z = 2), Wind (Z = 2), Net Radiation (Z = 2) and Soil Heat Flux (Z = − 0.05), where Z is height in meters in relation to an extensive surrounding surface of well-watered green grass [36]. Precipitation of rain and snow was measured hourly at 1.5 m and adjusted for wind effects [37]. The Makkink–Hansen estimation model was used to compare reference evapotranspiration with long-term on-site data [38]. A water balance simulation was made for each experiments using a plant and soil model DAISY and the agrometeorological data (For parametrisation of water balance model, see Additional file 1). Compartmentalized subsurface irrigation, intended to keep VWC above a threshold target of 20% (pF = 1.8), was initiated when the VWC sensors detected root water uptake within a compartment.

### Root measurements

Root imaging was conducted twice in each season at heading and at late anthesis (flowering). The root data presented in this paper were collected using RGB images following a grid intersection procedure [7]. Briefly, Images were collected for every 10 cm in 2016 and 5 cm in 2017 within the tube. The respective soil depth of each image was calculated as sin(horizontal angle in degrees) × tube depth + start depth. Each image covered an area of 0.05 × 0.035 m of the upper surface of the MR tubes and its interface with the soil. To determine the number of root intersections, a counting grid with a total line length of 0.33 m was superimposed on the image. In the second season, only white living root structures were recorded in order to avoid including still-visible root residue from the previous crop. The values were standardized as root intensity expressed as the sum of counts per m of gridline. Root intensity was averaged per 0.25 m of soil depth interval to represent the average root intensity at each depth and date in both seasons. For statistical analysis of differences between cultivars, deep root intensity was defined as the total number of intersections observed below 0.9 cm soil depth.

### Data analysis

The effect of position and cultivar on aboveground biomass measurements was tested using a TWO-WAY linear model with generalized least square (GLS), including errors in a Gaussian correlation structure based on the position in each unit (x,y). A similar model was used to test the effect of cultivar on deep root intensity. Here, the correlation structure was made in one direction based on the tube number. All tests were made using the nlme package in the software R [39]. For 2017 data, the effect of the unit was tested as a TWO-WAY Anova, including cultivar as fixed effect (Additional file 3). Estimated means of position and cultivars was determined using the emmeans package in R [40].

## Results

### Weather and growth conditions

**Fig. 5:**
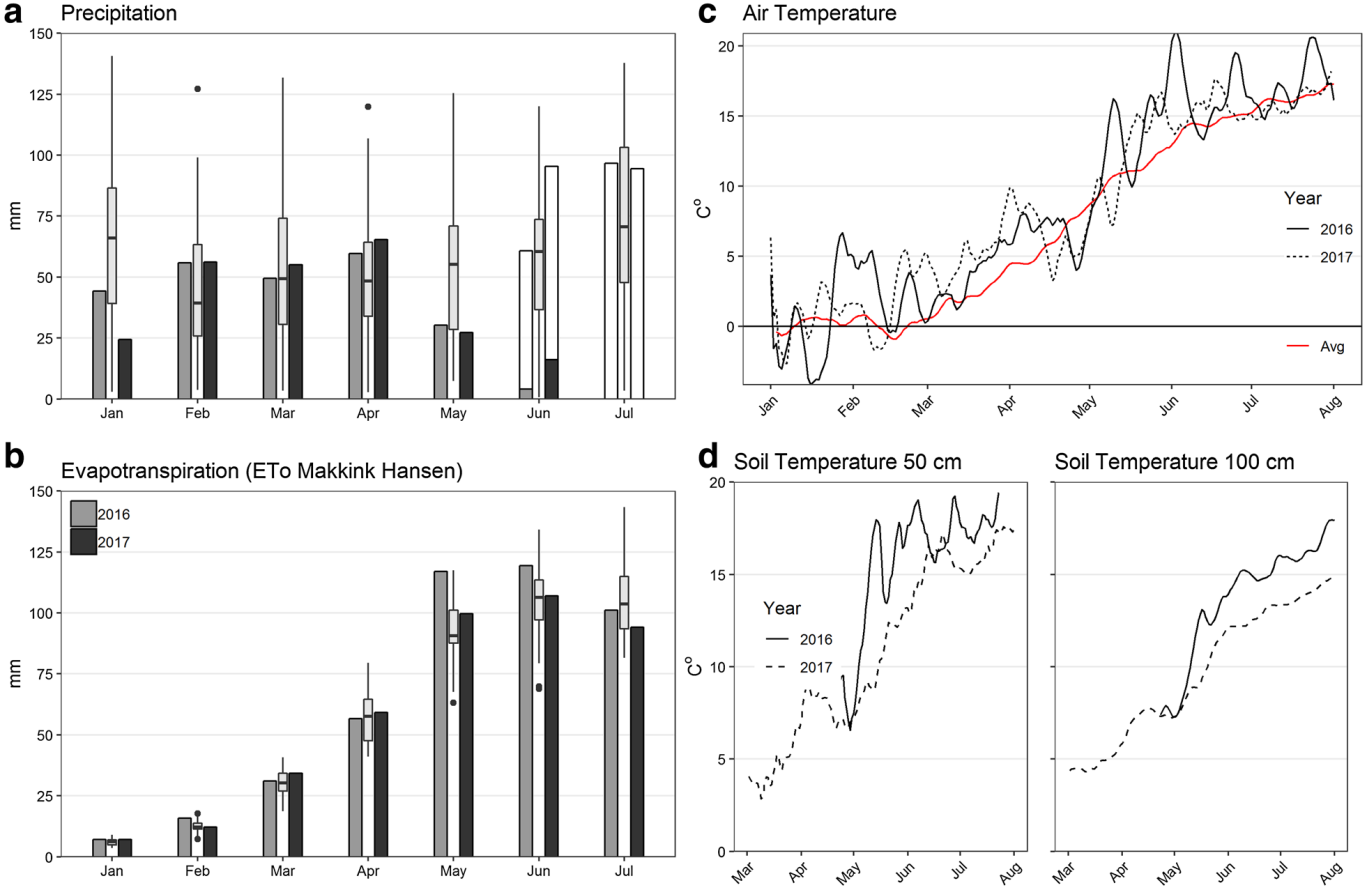
Weather data for two seasons of spring barley experiments. **a** Grey bars show the actual precipitation falling on the experiments, while white open bars in June and July indicate potential precipitation if rainout shelters had not been used. **b** Estimated reference evapotranspiration (Makkink–Hansen evapotranspiration estimates). **a** and **b** Solid bars show experimental data in both seasons, while boxplots relate to seasonal averages data from 1962 to 2008. Boxes show first quartile, median and third quartile. Extreme values are shown as points, while whiskers extend to the most extreme value within 1.5 × box length. **c** Lines represent daily air temperature as a 5-day moving average with the seasonal daily averages shown as a solid red line. **d** Daily soil temperature (0.5 m soil depth and 1.0 m soil depth)

**Fig. 6:**
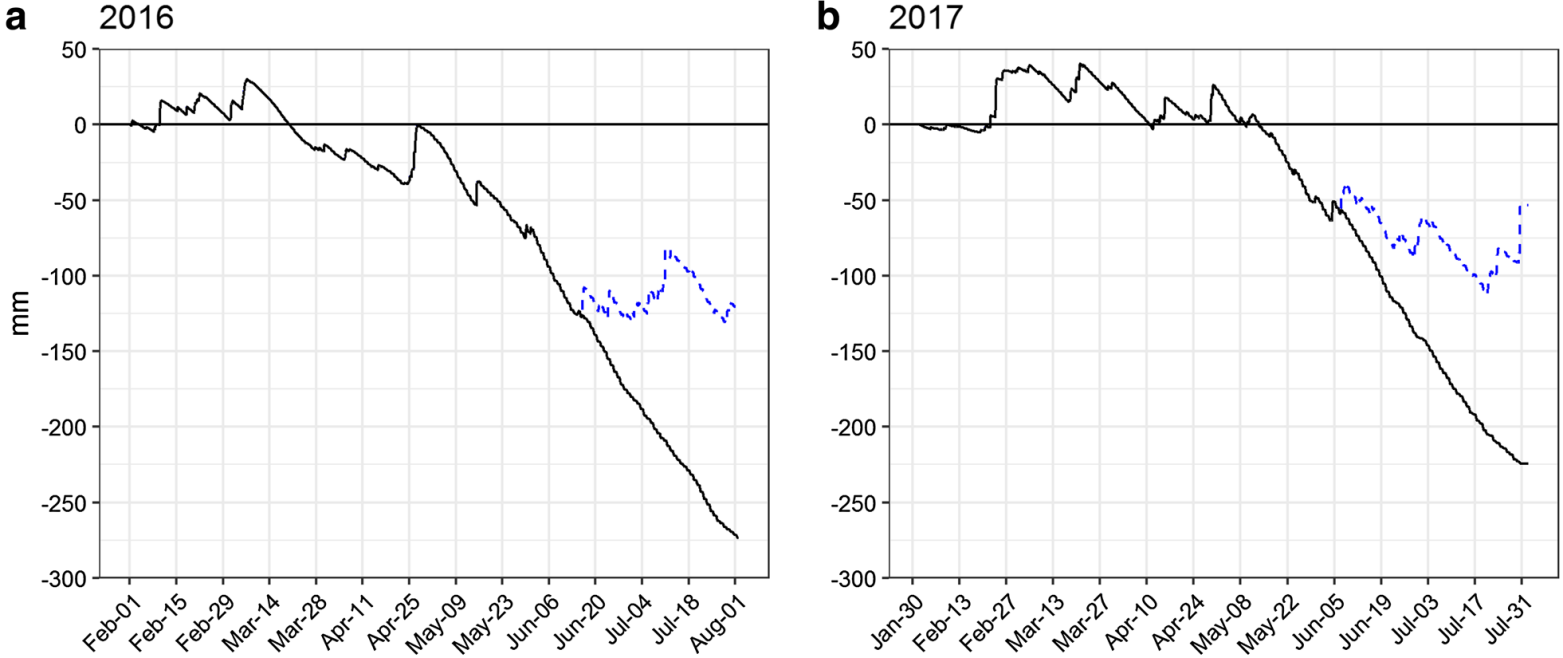
Simulated water balance of two spring barley experiments conducted in 2016 (**a**) and 2017 (**b**). The model was parameterised with the soil physical data from the topsoil (0 m–0.4 m) and subsoil (0.4 m–2 m) (solid black line). Spring barley managed as described in Table 2 with simulated drainage and potential evapotranspiration (see Additional file 1). The blue dashed line represents the water balance if no drought had been induced by rainout shelters

**Fig. 7:**
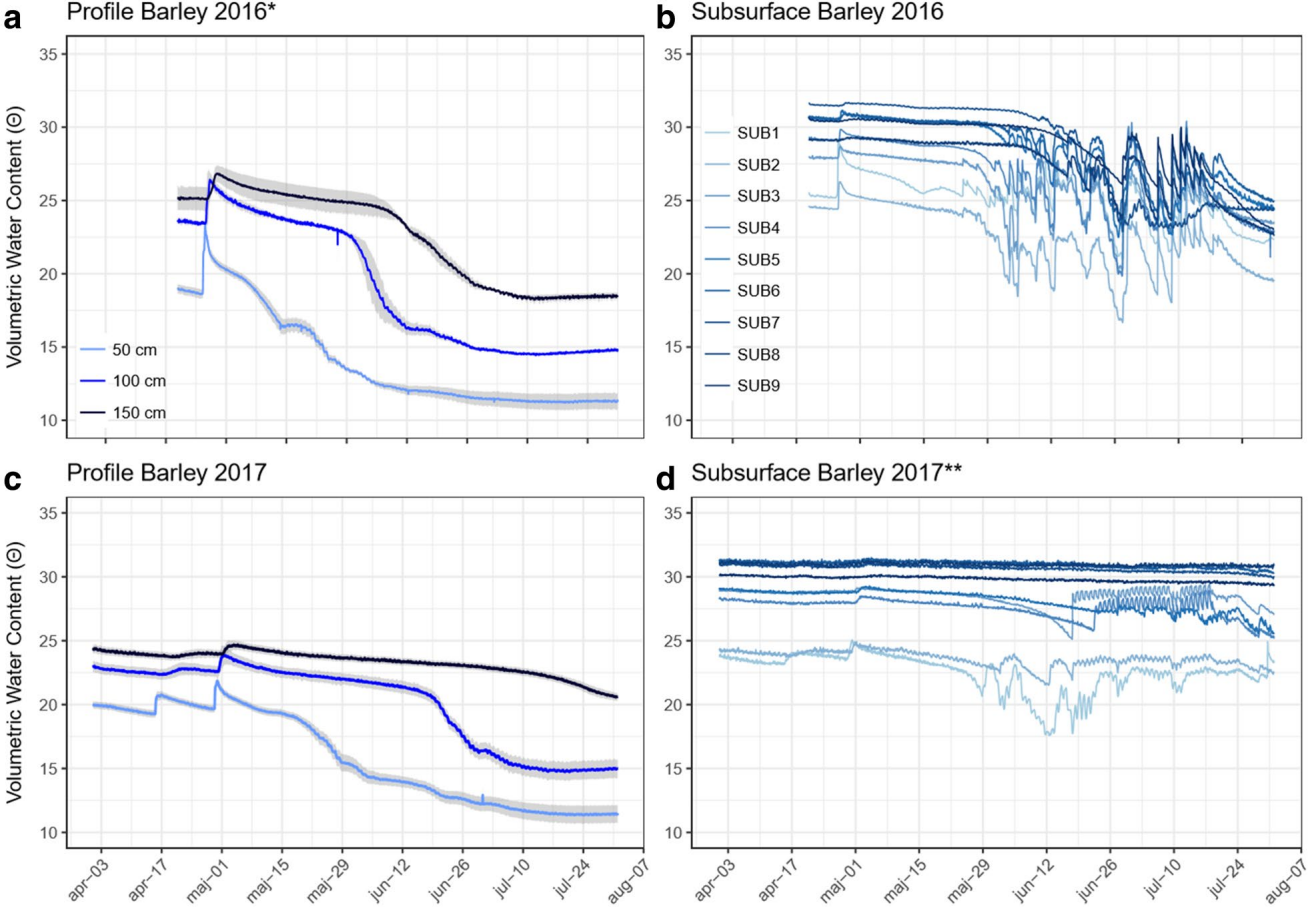
Volumetric water content (VWC) measurements from two experiments of spring barley. The subplots to the left (**a**, **c**) represent the mean VWC of three soil layers in the middle of each bed and the corresponding standard error, shown as a blue shade (n_50cm_ = 8, n_100cm_ = 4, n_150cm_ = 4). Subplots **b** and **d** show VWC data from the 9 subsurface irrigation compartments from the top section (SUB1, light blue) to the bottom section (SUB9, dark blue). *The 2016 experiment was made in one unit only (n_50cm_ = 4, n_100cm_ = 2, n_150cm_ = 2). **The 2017 experiment was made in units 1 and 2, while the 2016 experiments were made in unit 3. The depths and slope angle of the subsurface irrigation compartments are different

Both seasons were warmer than average, except for a cold period in late April (Fig. 5). May and June 2016 were exceptionally sunny, with two warm peaks in early and late May leading to a rapid increase in soil temperature and evapotranspiration (Fig. 5). Average levels of precipitation were measured in March and April, while May experienced dry conditions (< 25 mm precipitation) in both seasons. During the period from 1 February until the onset of the drought treatment by rainout shelters in June the precipitation was similar in the 2 years (2016; 199 mm, 2017; 220 mm). However, slight differences in rainfall pattern and drainage flow, combined with greater evapotranspiration rates in 2016, made the water deficit by early June 50 mm larger compared to 2017 when drainage and potential evapotranspiration (ET_c_) was simulated (Fig. 6). In both seasons, the water balance model indicates that precipitation in late April caused drainage of excess soil water to continue until early May, which is consistent with an observed peak seen for all soil water content sensors at 150 cm at this time point in both seasons (Fig. 7).

### Soil moisture measurements

The VWC sensors provided a precise measure of soil moisture with limited sensor-to-sensor variation, returning a high degree of precision having a low standard error (Fig. 7a, c). The data collected from the VWC sensor network yielded valuable information as to how water was depleted from the soil by the growing barley crop. In both seasons, the soil layer around 0.5 m experienced dry conditions (pF > 3.0 ≈ vol% < 15.3) from the beginning of June and soil water was rapidly depleted from the layer (Fig. 7a, c). Overall, more water was used in the 2016 season with a higher uptake even at 1.5 m than in 2017. In addition, soil water depletion at 1 m occurred approximately 3 weeks later in 2017 than in 2016, but in both seasons the soil ultimately reached the dry range of the retention curve. Importantly, subsurface irrigation by capillary rise was sufficient to keep the soil water content above 20% within the lowest irrigation compartments along the sloping bottom (see Fig. 7b, d), although soil in the midsection became dry below 1 m.

The experiments in 2016 were made in unit 3, and 2017 experiment was conducted in the deepest units 1 and 2. Consequently, the depths and slope angle of the subsurface irrigation compartments are different and not directly comparable. However, in 2016 a decline in soil water content were observed down to the deepest irrigation compartment at 1.83 m. In contrast, only the four upper sub-irrigation compartments showed signs of significant water depletion in 2017 (down to 1.61 m). In 2016, driplines were left running for long intervals that were followed by longer drying periods, leading to larger fluxes in soil water content. In 2017, subsurface water was emitted by a daily irrigation sequence of ten 5-min pulses over 10 h. The new irrigation strategy in 2017 resulted in more stable soil water content readings by the subsurface irrigation sensors (Fig. 7d).

### Root growth and development

**Fig. 8:**
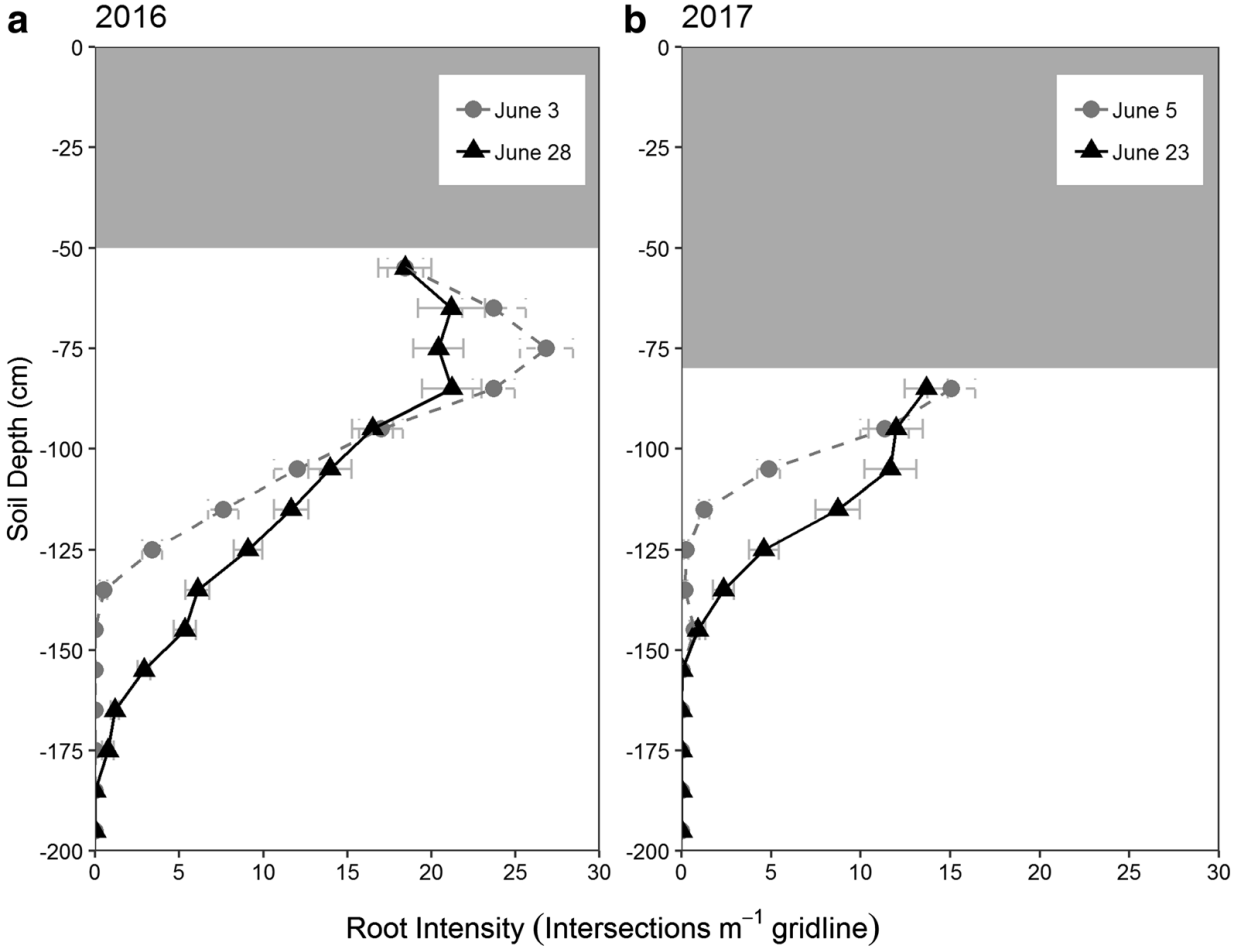
Depth distribution of root intensity. The figure shows the overall root intensity determined in 0.1 m increments for the two experiments of spring barley in 2016 (**a**) and 2017 (**b**) (mean ± standard error n = 28). Grey shaded areas in the top of the plot indicate soil layers not covered by the MR tubes. The experiment was conducted in units 1 and 2 in 2017, with root observations starting at 80 cm versus 50 cm for unit 3 in 2016

**Fig. 9:**
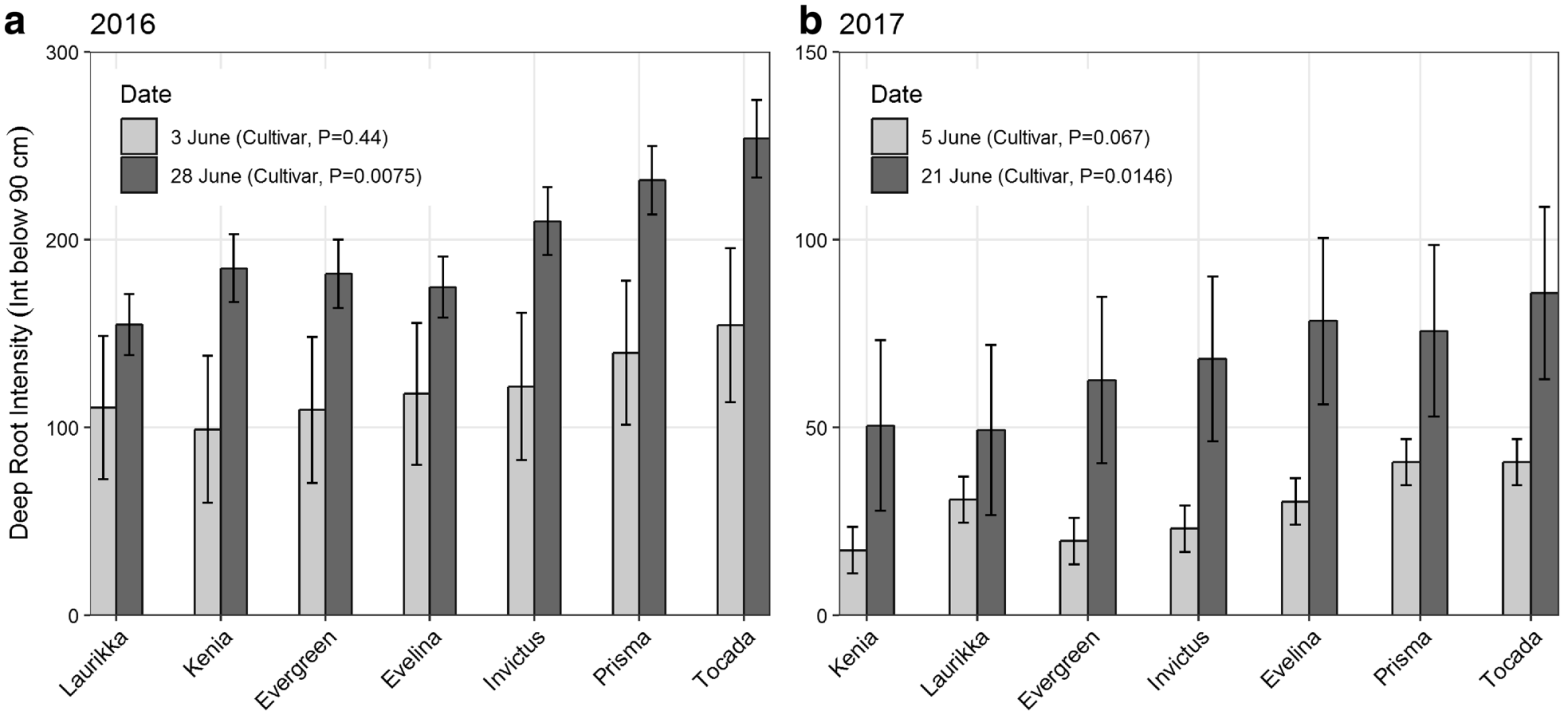
Deep root intensity of seven spring barley cultivars in the 2016 and 2017 seasons (**a**, **b**). Estimated mean and standard error n = 4. P values for cultivar effect in figure legend (one-way ANOVA.GLS model)

Roots grew deeper in the 2016 season than in 2017 (Fig. 8), which is consistent with a stronger decline of soil water at 1.5 m in 2016 (Fig. 8). Despite the high variation within MR data, significant differences among cultivars were found in both seasons (Fig. 9). Kenia and Laurikka consistently had fewer deep roots than Tocada and Prisma. A two-way ANOVA revealed a significant difference between the two units in the 2017 season (Additional file 3). Consequently, unit effects were included in the statistical model of the 2017 season.

### Effect of drought and subsurface irrigation on plant growth

Despite the dry conditions during anthesis and grain filling, the spring barley grain yield were only slightly reduced compared to regional average yield levels in both seasons. A low but significant effect of position on grain yield were seen in both seasons, with the highest yield level seen in the border section (Table 3). Also, a strong significant position effect was found for kernel size in both seasons, with kernels in the midsection being smaller than the border section. Grain in the midsection contained more protein in both seasons, but grain yield and estimated grain nitrogen removal (N-grain) was higher in the 2017 season than in the 2016 season.

**Table 3:** Comparison of the harvest samples of spring barley cultivars from the border and midsection of each unit in two seasons

	Grain yield		TKW		Protein		N grain	
	Mg ha^−1^		g		%		kg N ha^−1^	
	Border	Mid	Border	Mid	Border	Mid	Border	Mid
2016	6.03 (0.05)	5.56 (0.05)	52.0 (0.14)	51.0 (0.14)	7.9 (0.06)	8.10 (0.07)	74.9 (2.1)	70.6 (1.72)
Position	P < 0.0001		P < 0.0001		P = 0.044		P = 0.0029	
Cultivar	P < 0.0001		P < 0.0001		P < 0.0001		P < 0.0001	
Position × cultivar	ns; P = 0.32		ns; P = 0.98		ns, P = 0.40		ns; P = 0.26	
2017	7.22 (0.07)	6.98 (0.08)	53.6 (0.33)	52.6 (0.42)	9.30 (0.08)	9.60 (0.09)	107.5 (1.77)	107.3 (2.09)
Position	P = 0.0081		P = 0.0034		P = 0.0044		ns, P = 0.95	
Cultivar	P < 0.0001		P < 0.0001		P < 0.0001		P < 0.0001	
Position × cultivar	ns; P = 0.43		ns; P = 0.89		ns; P = 0.12		ns; P = 0.50	

Estimated means, standard error (in brackets) and P values from the corresponding two-way ANOVA output, (n(2016) = 4 × 59; n(2017) = 8 × 79). Grain yield, thousand kernel weight (TKW), protein content (Protein %) and estimated grain nitrogen removal (N grain)

Furthermore, a larger reduction in grain-yield and N-grain was observed in the 2016 season. In both seasons and for all aboveground parameters, the strong significant cultivar effect shows that it was possible to detect cultivar differences despite using a rather small sample size in the facility compared to the plot size normally used in field experiments. Other methods to detect the water stress gradient were tested in the 2017 season (Fig. 11). A visible increase in leaf temperature in the midsection was observed during the early grain filling using an airborne thermal camera. Similarly, a significant change in the carbon isotope discrimination ratio was found when comparing samples from the border section to the midsection.

## Discussion

### The minirhizotron phenotyping system

**Fig. 10:**
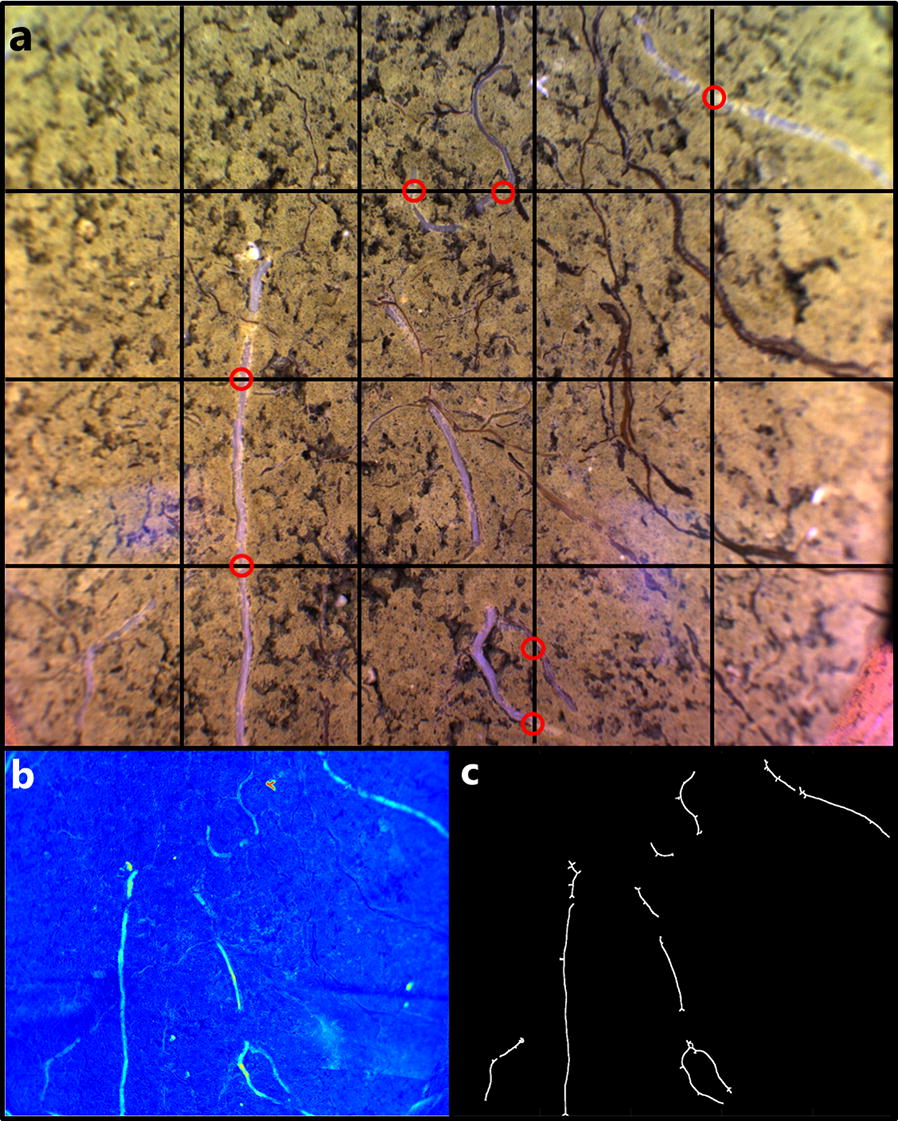
Minirhizotron root image of spring barley roots appearing with the presence of dead roots of red fescue with root length quantified by grid counting and by automated multispectral image analysis. **a** RGB type image with a grid structure applied for root quantification as performed in this study. Manual observers counted the number of times living white spring barley roots intersected with superimposed gridlines (red circles). **b** Transformed image using normalized canonical discriminant analysis (nCDA) **c** Final centerline extracted root length after noise removal using a vesselness enhancement filter

During construction of the facility, several measures were taken to improve the root phenotyping data delivered by MR-imaging. All 600 tubes were successfully installed during dry conditions, thus preventing mud smearing of the tube surface. In addition, since the MR tubes were not pushed into predrilled boreholes with tight soil connections, scratching along the tube surface was reduced. Furthermore, in contrast to field conditions where MR tubes are often installed with a slope angle greater than 45° [41, 42], our MR tubes were installed at an angle closer to horizontal (15.8°–23.5°). The low slope angle not only reduces the risk of roots growing downwards along the tube surface, but also provides a larger observation area per unit of soil depth covered. Finally, the careful installation procedure resulted in a homogenous soil background, which improves the root quantifications by eye or by automated image analysis (Fig. 10).

Of special concern was the risk of identifying old roots from the previous season, as the MR tubes are installed permanently. To account for this challenge, a multispectral imaging system inspired by [43] was developed. The system uses a portable trolley system and four multispectral camera systems, thus allowing for multivariate image analysis using five wavebands (405 nm, 450 nm, 590 nm, 660 nm, 940 nm) (Fig. 10). Using multivariate grouping of pixels based on differences in reflectance and by suppression background noise by a vesselness enhancement filter automated detection were possible (S.F. Svane, E. B. Dam, J. M. Carstensen and K. Thorup-Kristensen, unpublished observation). Since the facility contains a total of 600 MR tubes corresponding to ≈ 95,000 images per imaging campaign, traditional manual root quantification from individual RGB images would be time-demanding. The potential time savings of automated image analysis for root quantification was therefore critical for the full exploitation of the facility. Nonetheless, the root quantification presented in this paper was performed by the manual grid intersection procedure since the multispectral imaging system was not available in the 2016 season.

### Minirhizotron root observations

Although having only a short growing period, the spring barley crop developed a deep root system, extending to soil layers below 1 m. Roots extending below 1 m is deep for spring barley, but have been observed in the field both by MR imaging and soil coring under similar soil and climate conditions [44, 45]. Interestingly, large seasonal differences were present, with both deeper roots and greater root intensity in the 2016 season. Many factors within the soil environment could contribute to such differences. Soil texture, soil structural and climatological conditions such as rainfall pattern are known to have a large impact on the development of deep roots in the field [44]. Furthermore, low subsoil temperature is known to limit the root elongation rate [46, 47]. Differences in soil compaction level may also account for the observed year-to-year effect [48, 49] and finally seasonal differences in soil nitrogen level could influence the lateral root elongation rate [50–52]. Of the factors listed, measurements of soil temperature were available and revealed large differences in soil temperature between 2016 and 2017. An increase of soil temperature from 7 to 13 °C at 0.5 m depth, as observed between May 2017 and May 2016, has been found to increase the rate of root extension by ≈ 100% and the dry root weight of barley by 192% [47]. Measurements of soil bulk density and vertical distribution of soil nitrogen were not taken in 2017. However, it is likely that the soil was more compact and less nitrogen was present than in the 2016 season, the first year after construction. If so, these factors might also have contributed to the inter-year variation in root development.

Despite the observed seasonal differences in root development, a statistically significant effect of cultivar on deep root intensity was demonstrated. The cultivars Tocada and Prisma produced more roots in the deep soil layers in both years, and the ranking of deep root intensity of the seven cultivars was almost identical from 1 year to the next. However, no statistically significant differences among the cultivars were found in the early June comparisons of the 2 years, likely because of the low root counts in the early imaging interval decreasing the precision of the measurements.

Although significant cultivar differences were identified, the observed decrease in precision of the 2017 data may be explained by the observed block effect between units 1 and 2 (Additional file 3), which may be inferred from different pre-crops between unit 1 (wheat) and unit 2 (grass), leading to a difference in soil nitrogen levels. Such differences will be avoided by appropriate long-term planning in future studies within the facility.

Finally, the system was designed under the expectation that a distance between the MR tubes of 0.25 m is sufficient to exclude roots from neighboring rows. Currently the scale of this a potential neighbor effect is not known but a recent study have been made as an attempt to quantify the error (S. Chen, S.F. Svane and K. Thorup-Kristensen, unpublished observation). Here, water with ^15^N tracer was emitted along 15 MR tubes by pressure-compensated driplines. The tracer was only emitted along every second row and MR tube, which enabled investigation of ^15^N uptake by plants in unlabeled neighbor rows. It was found that plants in these neighboring rows contained only 10–25% of the ^15^N level found in target-row plants. As ^15^N will spread from the emission points, roots from neighbor rows can take up some ^15^N without growing all the way to the emission point. One way to more accurately determine the error term in a future study would be to MR-image plant lines with roots expressing a green fluorescent protein [53]. Despite the potential neighbor effect, a statistical significant cultivar effect was found in both seasons.

### Managing the water stress gradient

The control of a permanent water stress gradient using rainout shelters combined with a multi-depth subsurface irrigation system is a central part of the infrastructure. The subsurface irrigation system is designed to use the process of capillary rise to distribute water within each irrigation compartment. However, to accurately manage the water stress gradient it is important to know at which depth water uptake occurs. Information about the depth distribution of root water depletion is provided to some extent by MR imaging, and potentially provided by more advanced full-system crop models including a root growth model, e.g. [54, 55]. However, direct measurement of soil water by the VWC sensors is important in the daily management of the water stress gradient, as it allowed the startup of subsurface irrigation only when needed and thus saved unnecessary pumping activity in periods with no demand. The VWC sensors offered stable and precise measurements of soil water content. Low sensor–sensor variation, including similarly shaped curves during soil drying, indicates a similar porosity and soil texture within and between the units. This finding is supported by the low variation in soil texture and bulk density found by analyzing samples from auguring and soil coring between and within the units (Table 2 and Additional file 2).

The design with the sloping bottom combined with subsurface irrigation might risk horizontal water flow between the subsurface irrigation compartments. If present, the horizontal flow could transport nitrate-N from the border towards the midsection [56]. This effect would affect the interpretation of the stress symptoms in the aboveground leaves and the sampled biomass data, so bulkheads were installed to prevent horizontal water movement. Guided by the Mualem Van-Genutchen theory (Additional file 1), bulkheads were found to reduce maximum hydraulic conductivity from 10 cm h^−1^ in the saturated soil to 0.019 cm h^−1^ at the top of the bulkheads at a height of 0.25 m from the bottom. Furthermore, subsurface irrigation is initiated only when the soil water content starts to fall within the compartments, thus further reducing the potential for horizontal flow (Fig. 7b, d). Also, the observed biomass response with lower grain yield and smaller kernel size combined with the increase in protein level in the midsection reflects classical yield-protein relations during drought, e.g. [57], rather than the movement of N due to subsurface irrigation. Importantly, since all genotypes are grown in rows perpendicular to the gradient, similar differences in soil N status are expected among the genotypes tested.

### Detecting water stress

**Fig. 11:**
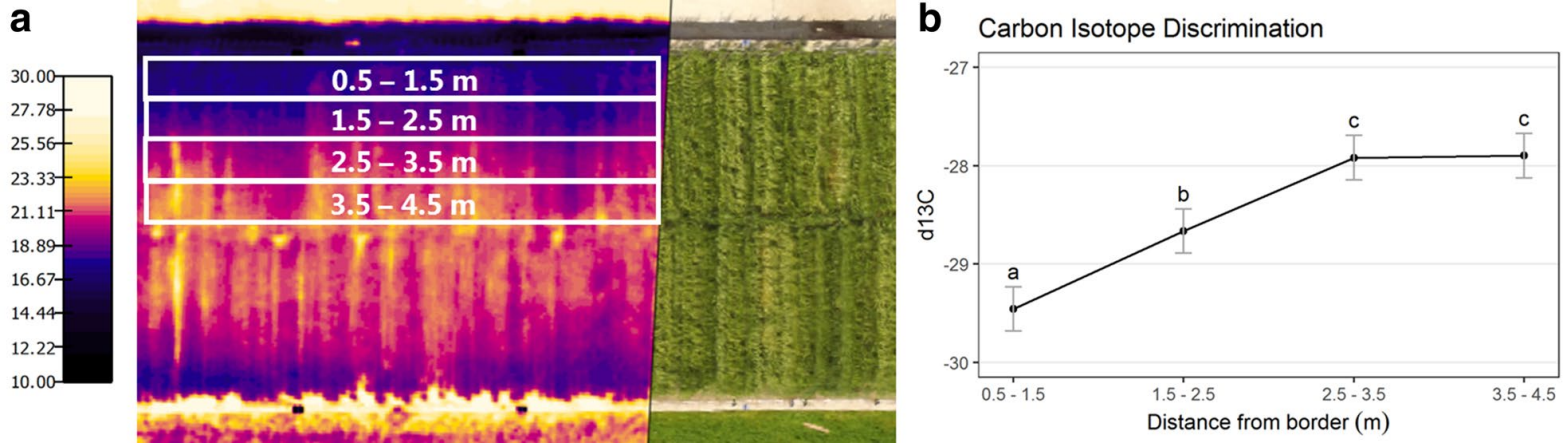
Suggested methods for water stress observations. **a** Thermal imaging from an unmanned aerial vehicle showing canopy temperature (°C), compared to RGB image of the crop and **b** carbon isotope discrimination ratio for the sampled area indicated on **a**

A novel element of this facility is the water-stress gradient created by rainout shelters and the subsurface irrigation system, inducing a visible response in the canopy. Current research observes drought symptom development in the canopy, using thermal and multispectral imaging (UAS-imaging), as well as manual scoring of drought symptoms (Fig. 11). This research also evaluates several other promising methods for quantifying differences between plant rows in temperature, leaf color or shape, each of which is a simple measure of how symptoms in individual genotypes develop over the distance from the border to the centerline of each unit. Of special interest is the use of thermal imaging, since leaf temperature is connected to changes in stomata conductance and is visible before any visible changes of form and color [58]. Such repeated and non-destructive measurements of the drought response in the canopy would provide valuable information for the detection of genotype performance across the gradient within the facility.

In both seasons, a statistically significant effect of position was observed. However, a sufficient available water content in the soil profile delayed the onset of drought symptoms until the grain-filling period. A similarly limited “drought effect” was seen in an lysimeter experiment using repacked soil to investigate drought tolearnce of spring sown wheat [59]. To stimulate the development of drought symptoms, improved management of the rainout shelters and the subsurface irrigation system could extend the period with dry conditions. Thus, a custom-designed drought treatments would improve the potential for detecting genotypic difference along the stress-gradient.

### The semi-field phenotyping approach

Large-scale semi-field setups have been widely used to understand soil hydrology, nutrient dynamics and plant water uptake, e.g. [19, 60, 61]. Results presented here confirm the premise that a semi-field setup offers several advantages over field experiments. Firstly, the construction process allowed for easy installation the MR-phenotyping system, VWC sensors and a subsurface irrigation system that would have been challenging and time-consuming to set up under field conditions. In addition, permanent control over precipitation and soil water allows for improved reproducibility of the drought treatment. Finally, since high levels of residual variation often hamper field experiments due to differences in soil texture and water [5, 62], in-field variation is reduced in a semi-field setup.

A similar reduction in field variation can be obtained by pot studies under controlled conditions. However, the use of a large soil volume with normal field nutrient levels avoids potentially confounding effects on plant physiology that are inherent to pot or container setups made with a smaller soil volume [63–66]. In addition, plants in the facility are exposed to natural levels of light, air temperature and, perhaps most importantly, soil temperature [67]. Nonetheless, the semi-field conditions within the facility do not address all variables that might be relevant to understand deep rooting under full field conditions. For instance, in the more compact subsoil found in fields, root growth could be restricted to biopores [44, 48, 49, 68, 69]. Also, constraints such as hypoxia, low soil temperature, soil acidity, aluminum and manganese toxicity, inadequate levels of calcium and phosphorus are known inhibitors for deep root growth in field conditions [46]. Of these, our facility provides low levels of phosphorus and suboptimal soil temperature in the early spring, compared to controlled greenhouse conditions. Furthermore, the soil bulk density of the subsoil is close to natural conditions of Danish subsoils of similar parent material (60 cm = 1.72 g cm^−3^) [70]. However, due to the excellent drainage conditions and high soil pH caused by the natural presence of carbonates, problems with hypoxia, calcium deficiency and metal related toxicity problems are not likely to be replicated in the facility. Thus, it would be valuable to validate results of experiments in our RadiMax facility under real field conditions, to identify potential genotype × environment interactions.

Finally, the current size of 2 × 150 rows per experiment is large compared to other research setups aimed at understanding and identifying phenotypic differences in deep root growth, but still limited compared to the requirements in genetic investigations (e.g. quantitative trait locus or genome-wide association studies). Instead, a combined approach can be made by performing a larger proxy trait screen under controlled conditions, with subsequent validation of a subset of contrasting genotypes within the facility as suggested by [5].

## Summary and conclusion

We successfully developed a new semi-field phenotyping system for the study of deep root growth and effective subsoil resource acquisition. The system offers a combined approach with direct measurements of root growth alongside detection of stress symptoms in the canopy, which represents a phenotyping through root function. A 2 year replicated spring barley experiment showed that phenotypic differences of deep root intensity could be repeated among spring barley cultivars and that clear aboveground physiological stress responses was visible along the water stress gradient.

The semi-field system offers labour-efficient and reproducible measurements of water and nutrient acquisition from deep soil layers of many genotypes compared to traditional field-based methods. Compared to in-house studies under controlled conditions, the semi-field condition enables the identification of relevant traits for full-scale crop development in an environment close to field conditions. In this way, we hope the semi-field facility concept will provide plant breeders with valuable data for the development of new robust and high yielding cultivars.

## Additional files

**Additional file 1.** Measurements of soil physical properties and parameterisation of water balance model.

**Additional file 2.** Texture and chemical properties of top and subsoil in each unit of the RadiMax facility (Fine soil fraction < 2 mm). The values are averages from two samples at each end of the unit, with the absolute difference in brackets. *Soil bulk density of the two sol layers measured at 0.25 m and 0.6 m in March 2017.

**Additional file 3.** Two-way ANOVA model output of biomass and root data from the 2017 season including the effect of the unit in the analysis.

## Abbreviations

ET_o_: reference evapotranspiration
ET_C_: potential evapotranspiration
MR: minirhizotrons
N: nitrogen
pF: soil water potential
TDR: trans domain reflectometry
TDT: trans domain transmission
VWC: soil volumetric water content
UAS: unmanned aerial system
