# Supplementary material for: Construction of a large-scale semi-field facility to study genotypic differences in deep root growth and resources acquisition
Source: Plant Methods. 2019 Mar 20;15:26. doi: 10.1186/s13007-019-0409-9 (PMC6425565; doi:10.1186/s13007-019-0409-9)

Construction of a Large-scale Semi-field Facility to Study Genetic Differences in Deep Root Growth and Resources Acquisition

Simon Fiil Svane[1], Christian Sig Jensen[2] and Kristian Thorup-Kristensen[1]
[1] *Department of Plant and Environmental Science, University of Copenhagen, DK-1871, Frederiksberg, Denmark*

[2] *DLF Seeds A/S, Research Division, DK-4660, Store Heddinge, Denmark*

Email:

Simon Fiil Svane: sfs@plen.ku.dk
Christian Sig Jensen: csj@dlf.com
Kristian Thorup-Kristensen: ktk@plen.ku.dk

Correspondence: Simon Fiil Svane, sfs@plen.ku.dk, +45 30293060

Meassurement of soil physical properties

Soil physical properties of the subsoil (soil cores from 0.6 m) were measured using the pressure plate and sandbed [1]. Suction was applied at eight points with different soil water potential, from pF 1 – pF 4.2. Soil water retention was measured in-situ using sensors carefully installed at field capacity. A small trench of 2 x 0.5 x 0.4 m (L x W x D) was excavated two weeks after germination of a spring barley crop in late April 2017. A VWC sensor True TDR-315 (Acclima, Inc., Boise, ID, USA), a T4 Tensiometer (UMS GmbH, Münich, Germany) and a matrix potential sensor MPS6 (Decagon devises, inc., Pullman, WA, USA) were inserted side-by-side into the undisturbed soil matrix to a final depth of 0.6 m, with a sensor-to-sensor distance of 0.1 m; tensiometers were installed with a 10° incline to the vertical line. In all, three replicated groups were made with a distance of 0.5 m within the trench. The soil was carefully backfilled, the spring barley seedlings replanted, and 25 mm of water added by a 30 min irrigation by a dripline system UniRam™ HNCL (Netafim, Tel Aviv, Israel). Data were collected by a CR1000 datalogger (CAMPBELL SCIENTIFIC, INC., UT, USA), with a 1 min sample interval. Water potential and content were recorded from pF 1.1 – pF 2. 93 by the T4 Tensiometer, while the potential at the dry end of the retention curve was measured by the MPS6 sensor pF 2.93 – pF 4.2. For each sensor group retention curves were fitted for each sensor position using a generalized additive model [2] using the software R (V. 3.5.1). Based on these three retention curves a single final subsoil retention curve was fitted using a local polynomial regression model (Fig. S1). Porosity was

calculated using the average bulk density and a particle mass density of 2.65 g cm^3. Field capacity was defined at pF 1.8 (Fig. S2).

Significant differences were observed between the retention curves measured in-situ and lab measurements made by pressure plates and oven drying, but only at water potentials larger than pF 2 (Fig, S3). Up to 4 vol %, more water was present in the soil cores in the laboratory at wilting point compared to the field. However, it must be noted that suction above field capacity (>pF 1.8) was applied by pressure plates in the laboratory, while suction in the field was applied by root water uptake driven by transpiration. Similar discrepancies have been seen in other studies comparing water retention curves determined by pressure plates to other methods, e.g., [3, 4]. Consequently, it was decided to use the in-situ measurements from the field to parametrize soil water retention curves for the subsoil. Recently, a similar strategy has been successfully applied in a facility developed to investigate root zone processes [5].

Water Balance Simulation

Water balance simulation was made using the mechanistic plant and soil model "Daisy" with water flow simulated by the Richards equation [6]. The simulation was made by parameterizing a 2 m reference soil column with drainage placed with a 50 cm lateral distance on top of an impermeable bottom. Parameterized topsoil physical properties (0 m - 0.4 m; Θ_{res}=2 %; Θ_{sat}=36 %; K_{sat}= 25 mm h^{-1}; α=0.0685 cm; n=1.30) were described by a Van genutchen model guided by Mualem theory with parameters obtained from topsoil bulk density and texture using the HYPRESS pedotransfer function [7]. Subsoil physical properties (0.4 m – 2 m; Θ_{res}=7.44 %; Θ_{sat}=35.3 %; K_{sat}= 10 cm h^{-1}; α=0.99 cm; n=1.19) were described using the in situ measured data points and the non-linear fitting program SWRC-FIT program using a Van Genutchen model. [8, 9]. No measure of saturated hydraulic conductivity (Ksat) was available. Instead, saturated hydraulic conductivity was calibrated to reflect sensor curves at 50 cm (Fig, S2). Potential evapotranspiration and drainage was simulated for the spring barley experiments in 2016 and 2017 (1. February to 2. of August). To compare the degree of soil drying between the two seasons, the FAO-Penman-Montieth (ET_o) was used as input for the simulation of potential evapotranspiration (ET_C) from the barley crop. ET_c was modeled using the standard Spring Barley development module in DAISY and assuming no limitation from lack of nitrogen and water. The model was parameterised with Soil preperation, Fertilization and sowing dates as presented in Table 2. Simulated ET_c and drainnage is presented in Fig. S4.

1. Dane JH, Hopmans JW. Water retention and storage: laboratory methods. In: Dane JH, Topp GC, editors. Methods of Soil Analysis: Part 4 Physical Methods. 5th edition. Madison, WI.: Soil Science Society of America; 2002. p. 671–720.

2. Wood SN. Fast stable restricted maximum likelihood and marginal likelihood estimation of semiparametric generalized linear models. J R Stat Soc Ser B Stat Methodol. 2011;73:3–36. doi:10.1111/j.1467-9868.2010.00749.x.

3. Solone R, Bittelli M, Tomei F, Morari F. Errors in water retention curves determined with pressure plates: Effects on the soil water balance. J Hydrol. 2012;470–471:65–74. doi:10.1016/j.jhydrol.2012.08.017.

4. Bittelli M, Flury M. Errors in Water Retention Curves Determined with Pressure Plates. Soil Sci Soc Am J. 2009;73:1453. doi:10.2136/sssaj2008.0082.

5. Cai G, Vanderborght J, Klotzsche A, van der Kruk J, Neumann J, Hermes N, et al. Construction of Minirhizotron Facilities for Investigating Root Zone Processes. Vadose Zo J. 2016;15:0. doi:10.2136/vzj2016.05.0043.

6. S. Hansen S, P. Abrahamsen P, C. T. Petersen CT, M. Styczen M. Daisy: Model Use, Calibration, and Validation. Trans ASABE. 2012;55:1317–35. doi:10.13031/2013.42244.

7. Wösten JHM, Lilly A, Nemes A, Le Bas C. Development and use of a database of hydraulic properties of European soils. Geoderma. 1999;90:169–85. doi:10.1016/S0016-7061(98)00132-3.

8. Seki K. SWRC fit - a nonlinear fitting program with a water retention curve for soils having unimodal and bimodal pore structure. Hydrol Earth Syst Sci Discuss. 2007;4:407–37. doi:10.5194/hessd-4-407-2007.

9. van Genuchtan M T. closed form equation for predicting the hydraulic conductivity of unsaturated soils. Soil Sci Soc Am. 1980;44:892–8. https://eurekamag.com/research/004/556/004556517.php. Accessed 13 Sep 2018.

Fig S1. In-situ measured retention properties of the subsoil at 60 cm. a) Three curves fitted to sensor measurements from three positions using a generalized additive model. Original measured values are shown as grey dots (data points = 85155). b) Predicted In-situ measured retention curve by polynomial regression model (span=0.2) based on data points from the three curves in A (n=240). Grey shade indicates a 95 % confidence interval of the predicted curve.

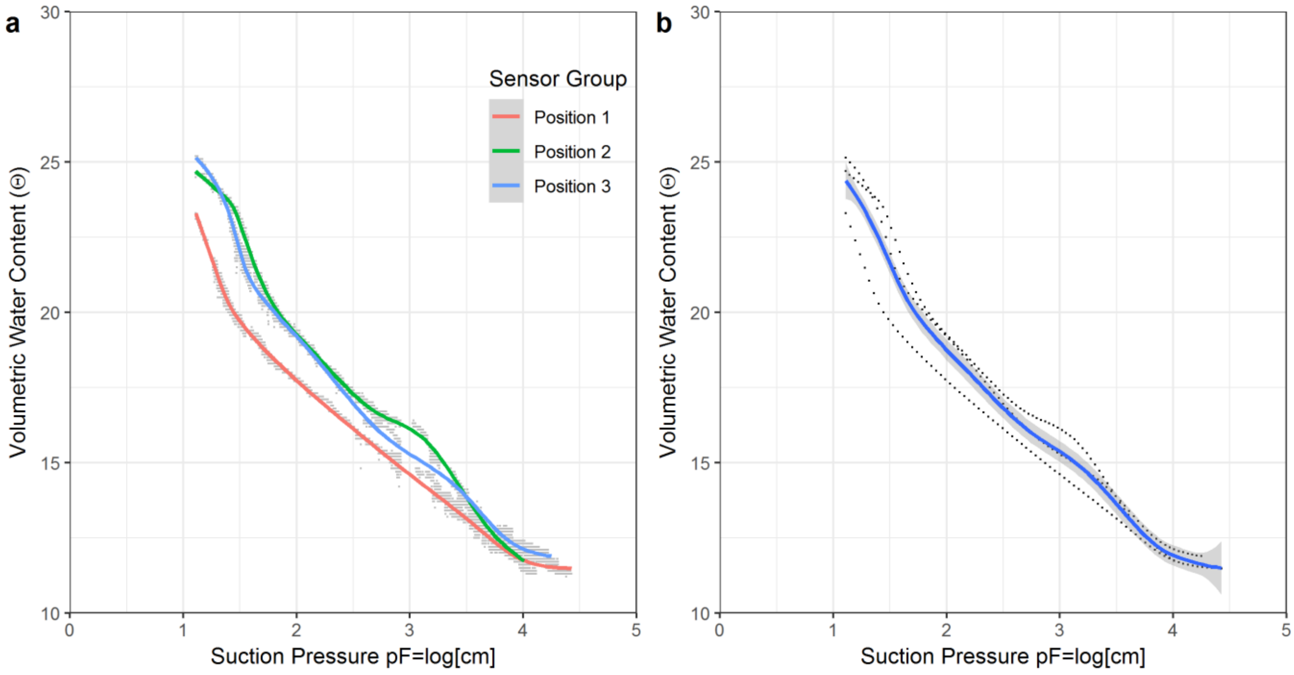

Fig. S2. Soil water release curves and simulated soil water content a) Van Genutchen water retention curves off topsoil determined by Hypress pefotransfer function and of the subsoil by fitting to measured data points at 60 cm (black dots, n=81). Original data points shown as grey dots. **b)** Measured volumetric water content at 50 cm as a solid line (mean value with standard error as grey shade, n=8). The solid blue line is simulated water content at 50 cm during drainage situations in early spring by the DAISY model.

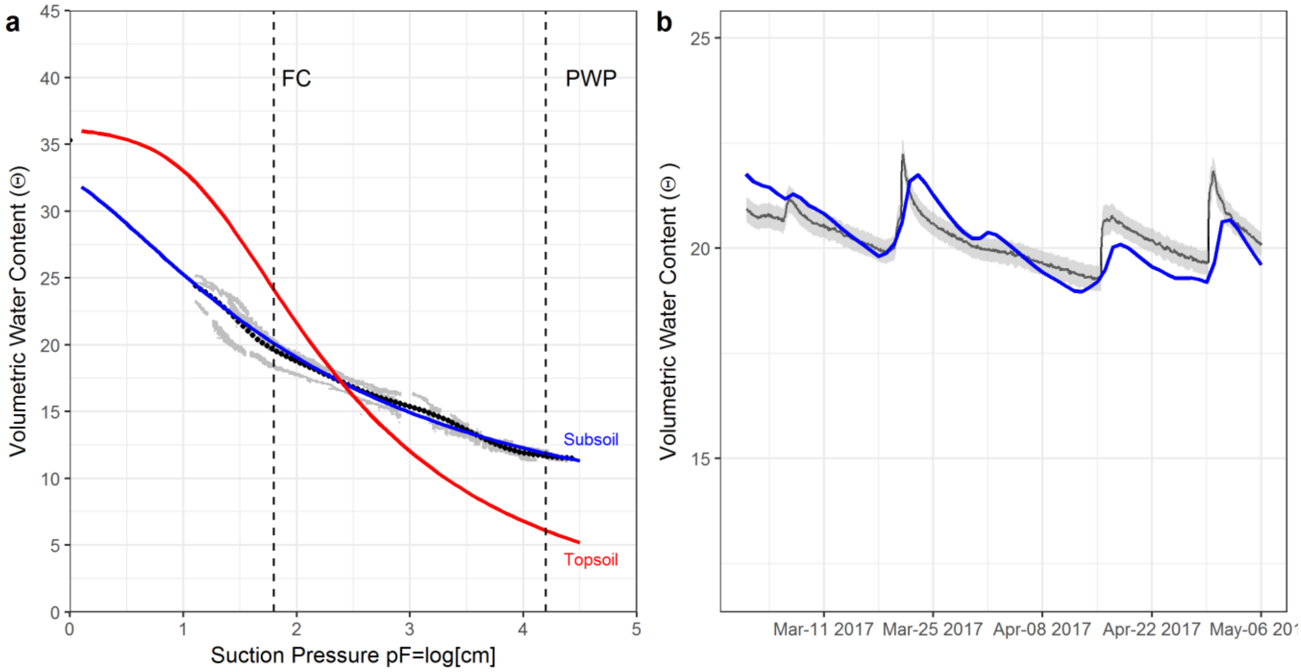

Fig. S3. Subsoil water retention at 60 cm. VWC is measured by TDR sensors using the Topp equation and by soil coring at seven different water potentials, followed by soil drying in the laboratory. Suction was applied by evapotranspiration in the field and by sand-bed (pF 1.3 – pF 1.7) and pressure plates (pF>1.7) in the laboratory. Mean values with ± 95 % confidence interval (Laboratory n=8, Field n=3). One-way ANOVA results for each group shown with black labels (***=P<0.001; **=P<0.01,*=P<0.05, · =P<0.1, ns (not significant)=P>0.1).

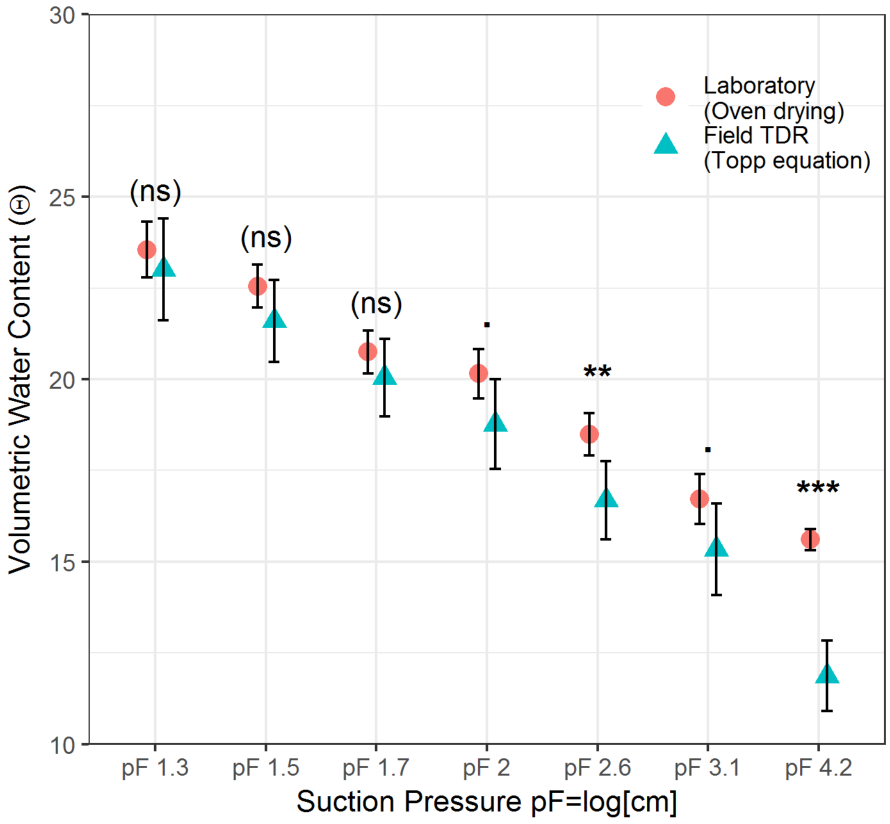

Fig. S4. Measured cumulated precipitation (solid black line) and simulated cumulated drainage (dashed blue line) and cumulated potential evapotranspiration of a barley crop (P_Evap, dashed red line) assuming no lack of water and nitrogen in two seasons. The dashed black line indicates precipitation received if the rain-out shelters had not been used.

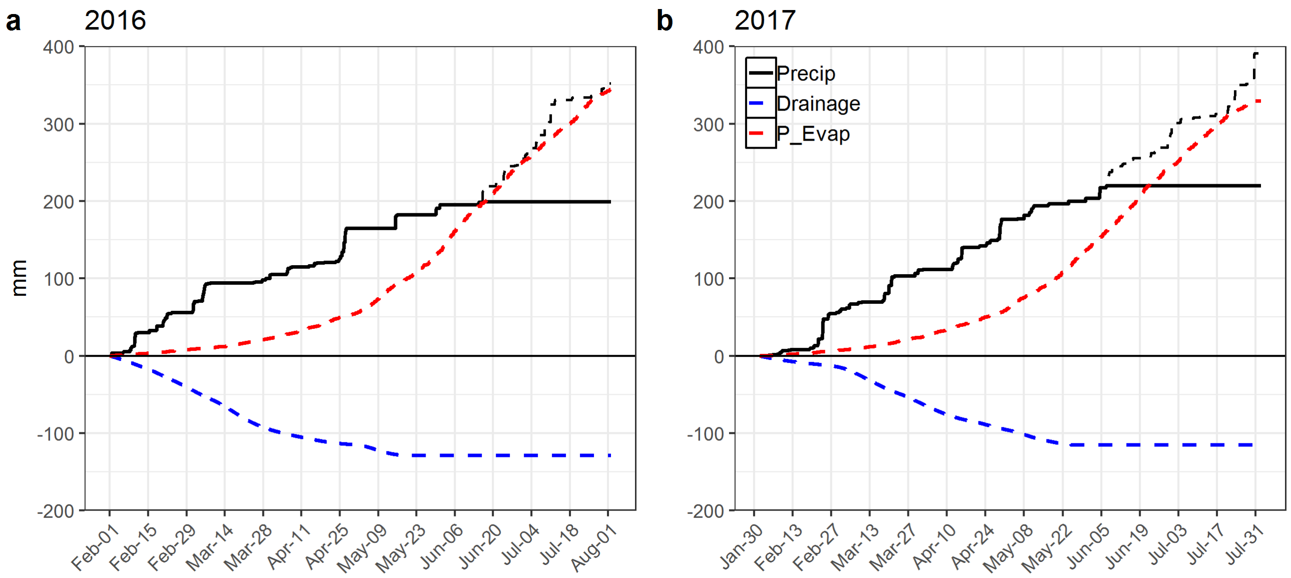

Supplement: Supplementary file 1 — Additional file 1. Measurements of soil physical properties and parameterisation of water balance model. [file 13007_2019_409_MOESM1_ESM.pdf]
